# Cognition, mood and quality-of-life outcomes among low literacy adults living with epilepsy in rural Kenya: A preliminary study

**DOI:** 10.1016/j.yebeh.2018.05.032

**Published:** 2018-08

**Authors:** Patrick N. Mwangala, Symon M. Kariuki, Moses K. Nyongesa, Paul Mwangi, Esther Chongwo, Charles R. Newton, Amina Abubakar

**Affiliations:** a Neuroassessment Group, KEMRI-Wellcome Trust Research Programme, Center for Geographic Medicine Research (Coast), Kilifi, Kenya; b Department of Public Health, Pwani University, Kilifi, Kenya; c Department of Psychiatry, University of Oxford, Oxford, UK

**Keywords:** Epilepsy, Adults, Neurocognitive impairment, Depressive symptoms, Quality of life, Kenya

## Abstract

Epilepsy is frequently associated with neurocognitive impairments, mental health, and psychosocial problems but these are rarely documented in low- and middle-income countries. The aim of this study was to examine the neurocognitive outcomes, depressive symptoms, and psychosocial adjustments of people with epilepsy (PWE) in Kilifi, Kenya. We evaluated the impact of these outcomes on health-related quality of life. Self-report, interviewer-administered measures of depression (Major Depression Inventory) and quality of life (RAND SF-36) were administered to 63 PWE and 83 community controls. Neurocognitive functioning was assessed using Raven's Standard Progressive Matrices, Digit Span, and Contingency Naming Test. The results show that PWE have poorer scores for executive function, working memory, intelligence quotient (IQ), depression, and quality of life than controls. Twenty-seven (27%) of PWE had depressive symptoms, which was significantly greater than in controls (6%); *P* < 0.001. Quality-of-life scores were significantly lower in PWE with depressive symptoms than in those without depressive symptoms (Mean QoL scores (standard deviation (SD)): 46.43 (13.27) versus 64.18 (17.69); *P* = 0.01. On adjusted linear regression models, depression affected total quality-of-life scores (*P* = 0.07) as well as individual health indicator domains touching on pain (*P* = 0.04), lethargy/fatigue (*P* = 0.01), and emotional well-being (*P* = 0.02). Our results show that epilepsy is associated with a significant burden of mental health and neurocognitive impairments in the community; however, community-based studies are needed to provide precise estimates of these disorders.

## Highlights

• Neurocognitive outcomes, depressive symptoms and quality of life among adults with epilepsy (PWE) were studied.
• Depressive symptoms were reported in 27% of PWE and in 6% of the controls.
• PWE had poorer scores for executive function, working memory, IQ and quality of life when compared to controls.
• Quality of life scores were significantly lower in PWE with depressive symptoms than in those without depressive symptoms.
• Epilepsy contributes to the burden of mental health and neurocognitive impairments in rural Kilifi.

## 1 Introduction

Epilepsy, a common neurological disorder, accounts for a significantly high proportion of the global burden of disease [1]. Approximately 70 million people globally are living with the condition; 80% of which reside in low- and middle-income countries [2], [3]. The prevalence of epilepsy is higher in sub-Saharan Africa (SSA) than in other parts of the world although its estimates vary across regions [4]. In Kilifi, the prevalence of active convulsive epilepsy is estimated to be 7.8/1000, but this could double if nonconvulsive epilepsy is taken into account [4]. Over two-thirds of epilepsy cases in Africa present with focal features [5], which are important risk factors for neurological impairments and cognitive and mental health disorders [5], [6], [7].

Several studies have shown that epilepsy is associated with multiple neurocognitive impairments [8], [9], [10], [11], [12]. Similarly, adults living with epilepsy have been observed to present with poor mental health and psychosocial adjustment problems including unemployment, poor marriage prospects, and limited education [10], [13], [14], [15], [16].

However, most of the existing research evidence arises from data from high-income countries. To the best of our knowledge, we do not know of any study that comprehensively investigates the neurocognitive and mental health outcomes of adults with epilepsy in SSA. It is likely that these outcomes are poorer in adults with epilepsy in malaria-endemic areas in SSA such as Kenya since neurocognitive impairments persist in children who have had cerebral malaria [17], which is a risk factor for epilepsy in this region [3], [17]. Data from other parts of the world cannot be extrapolated to guide interventions in Africa because of the differences in the proportion of symptomatic epilepsy, healthcare systems, access to medication, and formal and informal support systems among others. There is, therefore, an urgent need to investigate the long-term outcomes of epilepsy among adults in SSA.

We set out to conduct a comprehensive study examining the neurocognitive outcomes, depressive symptoms, and psychosocial adjustments of adults with epilepsy in Kilifi, Kenya. Specifically, we aimed to answer the following research questions:

1. Do adults with epilepsy experience neurocognitive impairments, depressive symptoms, and psychosocial maladjustments compared with age-matched controls in the community?
2. Do cognitive impairment and depressive symptoms in people with epilepsy (PWE) impact their health-related quality of life?

## 2 Materials and methods

### 2.1 Study site and population

This cross-sectional study was conducted at the Centre for Geographic Medical Research, Coast (CGMRC-Kilifi, Kenya), which has unique facilities for the study of neuropsychological functioning. Particularly, the center established the Kilifi Health & Demographic Surveillance System (KHDSS) in 2000 and currently conducts a census three times per year of a population of over 260,000, allowing for accurate follow-up of study participants [18]. Also, the CGMRC hosts an epilepsy clinic with a database of around 2000 people. Within this unit, we have technicians and an assessment team trained to administer measures of neurological, cognitive, and mental health functioning.

With a population of about 1.2 million people, 36% of the inhabitants of Kilifi County lack formal education, and only 52% have attained at least primary school level of education [19]. Majority of the residents are poor, earning a living either through subsistence farming or fishing [18]. The prevalence of epilepsy is high in the community [4]; about 62% do not access treatment [20].

### 2.2 Selection of study participants

This was part of a larger study conducted to evaluate the feasibility, acceptability, and reliability of using a mobile-based cognitive measure to assess and monitor chronically ill adults in Kilifi, Kenya. In this larger study, adults of low literacy were specifically targeted based on the assumption that they were more likely to experience challenges being assessed using mobile-based technology. It was envisaged that measures that can be used with the less literate populations will work with those who are better educated. As such, study participants were limited to only those with a primary level of education (up to class 8 in the current Kenyan education system).

#### 2.2.1 Participants living with epilepsy

Stratified random sampling was used to recruit 100 adults, based on standard psychometric guidelines where a sample size of at least 100 participants is needed for one to carry out internal consistency tests and get stable estimates [21]. Eligible participants were randomly selected from the Epilepsy database, followed in their rural homes, and included based on the following: i) if they had a confirmed epilepsy diagnosis; ii) they were aged 20 to 50 years; iii) they had no more than primary level of education; iv) they were able to provide informed consent together with a caregiver where needed; v) they were fluent in Swahili language; and vi) they had no symptoms of acute illness on the day of assessments.

#### 2.2.2 Control subjects

Controls were randomly selected from the KHDSS database, later approached at home, and requested to take part in the study. Those agreeing were referred to the clinic for assessment. All had to satisfy similar inclusion criteria as the cases apart from the epilepsy diagnosis.

### 2.3 Procedures

After giving consent, every participant underwent a detailed medical examination and neuropsychological assessment. Five-day training was given by the principal investigator, a research psychologist, to orient data collectors and supervisor on the measures to be used and the objectives of the study. Questionnaires were checked for completeness and consistency by the supervisor and principal investigator (Flowchart 1).

**Flowchart 1:**
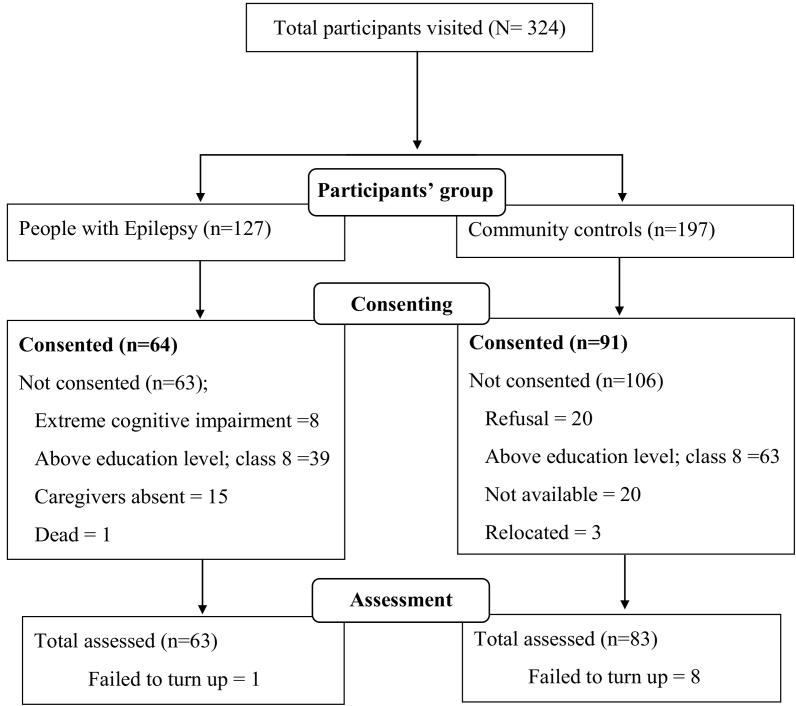
Summary of the recruitment process.

### 2.4 Measures

Consent forms, questionnaires, and tests were all translated to Swahili and back-translated and pretested before administration in the study.

#### 2.4.1 Cognitive functioning

Cognitive measures assessed three domains: executive functioning, working memory, and intelligence quotient (IQ). Three measures that have previously been used in Kilifi were administered: i) Raven's Standard Progressive Matrices (RSPM) [22], which is a nonverbal standardized measure of intelligence; ii) Contingency Naming Test (CNT), which taps the processing speed, attention shift, and response inhibition aspects of executive functioning and requires naming the color or shape of a series of stimuli according to different rules [23]; iii) Digit Span Backwards, which is a test of memory span having a list of random numbers which are read aloud to the participant who is then required to recall the items in a reverse order [24].

#### 2.4.2 Depressive symptoms

Major Depression Inventory (MDI) is a self-report measure of moods experienced in the past 2 weeks aimed at investigating depressive symptoms [25]. It has 12 items; however, during scoring, only 10 items were considered since items 8 (a) and (b) and items 10 (a) and (b) were collated into two items. The measure is scored on a 6-point Likert scale with ‘0’ being at no time and ‘5’ at all the time. As a severity measure, the tool's score ranges from 0 to 50. Mild depression was interpreted as an MDI total score of 20–24. Moderate depression was equated to an MDI total score of 25–29, and severe depression for scores of 30 or more. This measure has been adapted and yielded reliable psychometric properties among young adults in Kilifi [26].

#### 2.4.3 Quality of life

The RAND 36-Item Health Survey 1.0 is a generic measure of health-related quality of life. It assesses eight health domains namely physical functioning, bodily pain, role limitations due to physical health problems, role limitations due to personal or emotional problems, emotional well-being, social functioning, energy/fatigue, and general health perceptions [27]. Scoring the tool is a two-step process. Firstly, precoded numeric values are converted to percentages using a predetermined scoring key with a high score defining a more favorable state of health. Secondly, items in the same scale are averaged together to create the 8 different scale scores.

#### 2.4.4 Socioeconomic status (SES)

A 13-item asset index, which is used reliably to assess SES over time in studies conducted in this rural setting, was administered to adults with and without epilepsy [28], [29]. The asset index items screen for the ownership of a list of disposable assets by participants (or their family) such as radio, television, bicycle, and motorbike. A single index score of SES was generated, with a higher score indicating a higher SES.

### 2.5 Clinical characterization of PWE

Epilepsy-related features including semiology (focal versus generalized seizures), frequent versus infrequent seizures, electroencephalogram (EEG) recordings, and antiepileptic drug (AED) treatment were available for 51 participants. Complete case analysis involving the 51 PWE was carried out to evaluate the potential impact of these clinical characteristics on neurocognitive impairment, depressive symptoms, and quality of life.

### 2.6 Statistical analysis

Frequency, percentages, and median were used to describe demographic variables. Independent *t*-test and chi-square analyses (with Fisher's exact test where appropriate) were used to assess group differences in demographic characteristics. Univariable linear regression analysis was used to examine group differences in depressive symptom scores, cognitive test scores, as well as quality of life scores. Multivariable linear regression was then conducted to examine the impact of depression and cognitive outcomes on the quality of life adjusting for age, sex, SES, and level of education. Permission to conduct the study was granted by the institutional review board of KEMRI, SERU (KEMRI/SERU/CGMR-C/030/3187) and the Kilifi County Hospital. Written informed consent was obtained from the participants after the study had been fully explained to them.

## 3 Results

### 3.1 Descriptive statistics

Compared with controls, adults living with epilepsy were significantly younger, presenting with lower educational outcomes, and likely to be unmarried. Significant differences were also observed in the occupational status of the two groups (*P* = 0.05), with the group with epilepsy being more likely to be unemployed. Table 1 below summarizes the demographic characteristics of the two groups.

**Table 1:** Demographic characteristics.

	Epilepsy n (%)	Controls n (%)	*P* value
Age median (IQR)	27 (23.5–33.0)	33 (25–42)	**0.001**
Sex (female)	34 (54)	53 (63.9)	0.23
Education level			
None	15 (23.8)	8 (9.6)	**< 0.001**
Primary incomplete	40 (63.5)	26 (31.3)	
Primary complete	8 (12.7)	49 (59.0)	
Marital status			
Never married	53 (84.1)	20 (24.1)	**< 0.001**^a^
Separated	3 (4.8)	4 (4.8)	
Married	7 (11.1)	59 (71.1)	
Occupation			
Not employed	36 (57.1)	35 (42.2)	**0.05**
Self employed	27 (42.9)	43 (51.8)	
Professional	–	5 (6.0)	

a Based on Fisher's exact test.

### 3.2 Clinical characteristics of PWE (N = 51)

Majority of the respondents (82.1%) had lived with epilepsy for more than 15 years. A total of 20 (60.6%) PWE had abnormal EEG. In terms of medication, 31 (60.8%) were on AEDs. Forty-two (82.4%) had focal seizures, 12 (23.5%) presented with generalized seizures, while 27 (52.9%) exhibited both types of seizures. Table 2 below gives a summary of these characteristics.

**Table 2:** Clinical characteristics of PWE (N = 51).

Variable	n (%)
On antiepileptic drugs (AED)	31 (60.8)
Presenting with abnormal EEG	20 (60.6)
Presenting with focal seizures	42 (82.4)
Presenting with generalized seizures	12 (23.5)
Presenting with both focal and generalized seizures	27 (52.9)
Seizure frequency	
Daily	18 (35.3)
Weekly	21 (41.2)
Monthly	5 (9.8)
Yearly	7 (13.7)

### 3.3 Depressive symptoms in PWE vs. controls

A substantial number of PWE (34.9%) were not able to complete the MDI questionnaire as they were not able to follow instructions because of extreme cognitive impairment. In contrast, all the controls were able to complete the questionnaire. The results reported are from the participants who were able to complete the questionnaire. People with epilepsy had significantly higher depressive symptom scores. In both the univariable and multivariable analyses, there were significant differences between the PWE and controls in their depressive symptom scores (*P* < 0.001 and *P* = 0.01, respectively) with PWE presenting with worse outcomes (Table 3). Among PWE, 11 (27.0%) met the MDI's criteria for depression compared with 5 (6.0%) among the community controls.

**Table 3:** Depressive, cognitive, and quality-of-life score differences between cases and controls (crude and adjusted estimates).

	Unadjusted					Adjusted	
Variable	Cases		Controls		*P*-value	Adjusted coefficient (95% CI)	*P*-value
	Mean (SD)	Median	Mean (SD)	Median			
MDI scores (depressive scores)	13.4 (8.6)	13	8.0 (7.3)	7	**< 0.001**	4.8 (1.3–8.3)	**0.01**
CNT total errors	30.4 (10.2)	26	24.5 (8.4)	25	**0.002**	− 4.1 (1.4 to − 9.1)	**0.01**
Ravens total score	14.6 (6.2)	16	19.8 (7.3)	18	**< 0.001**	− 3.6 (− 6.7 to − 0.5)	**0.02**
Digit Span (highest score)	1.7 (2.4)	0	6.4 (3.3)	6	**< 0.001**	− 4.1 (− 5.3 to − 2.8)	**< 0.001**
Quality of life total score	59.4 (18.3)	57.4	78.1 (14.7)	80.4	**< 0.001**	− 20.5 (− 27.7 to − 13.4)	**< 0.001**
Physical functioning average score	75.1 (26.2)	80	94.4 (12.8)	100	**< 0.001**	− 20.2 (− 27.5 to − 12.9)	**< 0.001**
Role functioning/physical average score	49.4 (39.3)	50	77.7 (33.6)	100	**< 0.001**	− 29.5 (− 44.2 to − 14.9)	**< 0.001**
Role functioning/emotional average score	42.3 (46.6)	0	67.0 (43.7)	100	**0.003**	− 30.6 (− 50.9 to − 10.3)	**0.003**
Energy/fatigue average score	49.4 (22.8)	55	62.1 (19.9)	60	**0.01**	− 14.8 (− 24.3 to − 5.2)	**0.003**
Emotional well-being average score	59.4 (22.9)	56	71.2 (21.2)	76	**0.01**	− 13.3 (− 22.4 to − 4.2)	**0.01**
Social functioning average score	59.5 (22.8)	62.5	75.9 (20.9)	75	**< 0.001**	− 16.7 (− 27.0 to − 7.0)	**0.001**
Pain average score	53.6 (29.0)	55	71.9 (21.1)	70	**< 0.001**	− 20.5 (− 31.5 to − 9.5)	**< 0.001**
General health average score	55.0 (21.9)	50	77.1 (17.2)	80	**< 0.001**	− 24.1 (− 32.8 to − 15.4)	**< 0.001**

SD: standard deviation; CI: confidence interval.

### 3.4 Cognitive outcomes in PWE vs. controls

There were significant differences between PWE and controls in working memory scores, in both univariable analysis (*P* < 0.001) and multivariable analysis (*P* < 0.001) with PWE performing poorer (Table 3). This was also the case in the IQ scores in both univariable analysis (*P* < 0.001) and multivariable analysis (*P* = 0.02). A similar trend was observed in the executive functioning outcomes in univariable analysis (*P* = 0.002) and multivariable analysis (*P* = 0.01). For the IQ test, 20 (31.8%) PWE were unable to do the test compared with 23 (36.5%) in the executive functioning measure. A further 12 (19.1%) were not able to do the memory test among PWE. In all cases, participants with incomplete assessments were excluded from respective analyses.

### 3.5 Quality-of-life outcomes in PWE vs. controls

Adults living with epilepsy performed significantly worse compared with the controls in their total QoL scores in both univariable analysis (*P* < 0.001) and multivariable analysis (*P* < 0.001). When individual domains were compared, the scores from adults living with epilepsy were significantly poorer compared with those from controls across all the QoL domains as shown in Table 3.

### 3.6 The impact of depressive symptoms on the quality of life of PWE

People with epilepsy and depressive symptoms had lower QoL scores in all domains compared with those without depressive symptoms. In univariable analysis, PWE who have depressive symptoms had significantly lower scores in their total QoL scores, physical functioning, role functioning, energy/fatigue, emotional well-being, and general health domains with *P* < 0.05. In multivariable analysis, PWE who have depressive symptoms had significantly lower scores in energy/fatigue, emotional well-being, and pain domains with *P* < 0.05. Table 4 presents these findings.

**Table 4:** The impact of depressive symptoms on the quality of life (QoL) among PWE (crude and adjusted estimates).

	Unadjusted					Adjusted	
QoL Variable	Positive for depression		Negative for depression		*P*-value	Adjusted coefficient (95% CI)	*P*-value
	Mean (SD)	Median	Mean (SD)	Median			
Total quality-of-life score	46.4 (13.3)	45.7	64.2 (17.7)	64.8	**0.01**	− 12.6 (− 26.2 to 1.0)	0.07
Physical functioning average score	65.5 (26.4)	70.0	78.7 (25.7)	87.5	**0.03**	4.6 (− 14.0 to 23.3)	0.62
Role functioning/physical average score	27.3 (36.2)	0	57.5 (37.8)	62.5	**0.03**	− 20.2 (− 51.7 to 11.4)	0.20
Role functioning/emotional average score	30.3 (40.7)	0	46.7 (48.4)	33.3	0.41	− 15.0 (− 51.5 to 21.5)	0.41
Energy/fatigue average score	35.5 (21.8)	30.0	54.5 (21.2)	55.0	**0.02**	− 25.2 (− 43.8 to − 6.7)	**0.01**
Emotional well-being average score	43.6 (33.7)	40.0	65.2 (22.8)	64.0	**0.01**	− 22.0 (− 39.7 to − 4.3)	**0.02**
Social functioning average score	62.5 (20.9)	62.5	58.3 (23.8)	50.0	0.59	5.2 (− 15.9 to 26.4)	0.62
Pain average score	38.6 (30.7)	25.0	59.1 (26.8)	55.0	0.08	− 26.0 (− 50.3 to − 1.6)	**0.04**
General health average score	41.4 (21.3)	40.0	60.0 (20.2)	55.0	**0.01**	− 17.3 (− 35.9 to 1.2)	0.07

SD: standard deviation; CI: confidence interval.

### 3.7 The impact of epilepsy-related features on depressive symptoms, neurocognitive impairment, and quality of life among PWE (N = 51)

We found no evidence of an association between type of seizures (focal versus generalized seizures) and neurocognitive impairments, depressive symptoms, and quality of life (*P* > 0.1). Likewise, frequency of seizures was not associated with any of these outcomes (*P* > 0.1). Similarly, EEG abnormalities and AED treatment did not have an impact on neurocognitive impairment, depressive symptoms, and quality-of-life outcomes (*P* > 0.1).

### 3.8 The impact of cognitive impairment on the quality of life of PWE

We explored the impact of cognitive impairment on the quality of life of PWE. In this case, we defined cognitive deficit in the group with epilepsy as any cognitive performance that is below the third quartile (75th percentile) score in the control group. Using this criterion, we observed few significant associations between being cognitively impaired and quality of life. Cognitively impaired participants performed significantly worse in the social functioning domain in the IQ scores (*P* = 0.04) and the general health domain of their memory scores (*P* = 0.02). The results of this set of analysis are presented as Supplementary Results.

## 4 Discussion

This study was performed to understand if the associations of epilepsy with behavioral problems and neurocognitive problems observed in children from this area [30] occurred in adults with epilepsy. The results show that epilepsy in adults is associated with poor scores for executive function, working memory, IQ, depression, and quality of life. Twenty-seven percent (27%) of adults living with epilepsy had depressive symptoms, which was significantly greater than in controls (6%). The situation was worse than we had anticipated before the study since epilepsy was so severe in over 30% of adults that they could not complete tests for depression and neurocognitive impairments and were consequently excluded from respective analyses.

### 4.1 Depressive symptoms in adults with epilepsy

Both median and mean scores for depression assessed with Major Depression Inventory, which has been validated for use in Kilifi, were more frequent in PWE than in controls. These differences in mean scores are similar to those of behavioral/emotional problems that were observed in children with epilepsy from previous studies in this area [30]. It is possible that behavioral and emotional problems, which are common in children living with epilepsy in this area [31], can persist into adulthood as other mental health problems such as depression, but need to be confirmed through long-term cohort studies. Moreover, PWE were more socioeconomically deprived than the controls, a factor which is universally known to be associated with depression [32]. In view of this, we built regression models, adjusted for potential confounders including socioeconomic status, and found that depressive symptoms were still significantly associated with epilepsy in adults, with very large beta coefficients. This association between epilepsy and depression is recognized in the literature [33], but our findings are among the first to show this relationship in SSA. However, the association cannot determine whether epilepsy is either a cause or consequence of depression. There is research to indicate that mental health problems can be antecedents to development of epilepsy [34], [35], [36]. This study highlights the need to conduct epidemiological studies to estimate the burden of depression and other psychiatric problems in the community in addition to more carefully designed longitudinal studies to have a better understanding of the causal pathways.

### 4.2 Neurocognitive problems in adults with epilepsy

Adults with epilepsy made more errors in the contingency naming test and had the lowest scores in intelligence and memory tests. These findings remained significant even after accounting for sociodemographic confounders. The results may explain why a high proportion of PWE had learning and intellectual disabilities in this study (up to 48%) and in other community-based studies in this area [5]. Optimal cognitive functions are crucial in daily activities and would contribute substantially to the years lived with disability among adults living with epilepsy as was shown in our two previous studies [37], [38]. The extent of the cognitive problems in epilepsy in this study is even substantially greater than can be inferred from results from these cognitive tests, considering that 37% of the adults with epilepsy were unable to complete the executive function tests, and likely represents severe disability in PWE, who perhaps require special support and care including rehabilitation. Unfortunately, a recent situation analysis showed that such services are not available for people living with mental health problems in this area [39]. Although care for children with disabilities including rehabilitation services has been given some attention in this area [40], the focus should also be extended to the adults and the aging populations in whom disabilities are also common.

### 4.3 Quality of life in adults with epilepsy

Adults with epilepsy had significantly lower scores for quality of life than controls, with the associations remaining significant in adjusted models. Almost every aspect of life was affected including social/physical functioning, emotional well-being, and general health status. These findings are not surprising since epilepsy is associated with stigma especially in poor areas such as Kilifi [41] and, therefore, would impact emotional well-being and social/physical functioning of PWE. The findings are consistent with the significantly higher proportion of poor marriage prospects, unemployment, and illiteracy documented among PWE in this study, a finding which was also observed in samples drawn from a population-based study in this area [5]. The association with poor health status is very interesting and is probably related to the following: (i) poor nutrition observed in PWE from this region [5]; (ii) inability to access quality healthcare because of socioeconomic disadvantage from having epilepsy; or (iii) the enormous comorbidity burden seen in PWE compared with that in people with other chronic conditions. We attempted to answer the third possibility by examining the impact of having depression on the quality of life in adults with epilepsy and found that quality-of-life scores were significantly lower in those with depression compared with scores in those without depression among PWE. On adjusted models, depression affected quality of life particularly on the domains touching on pain (which is a health status indicator), lethargy/fatigue (a health status indicator), and emotional well-being. These results underline the need to address both psychosocial and medical needs in PWE.

We examined the impact of cognitive impairment on the quality of life in adults with epilepsy and found no evidence of an association between them. In contrast to our findings, earlier studies have reported that cognitive outcomes influence the quality of life of patients with epilepsy [42], [43], although these studies are from high-income countries. These differences may have arisen from other factors including medical comorbidities and nutritional status that may moderate the severity and patterns of cognitive impairment to influence the quality of life in adults with epilepsy in our setting but were not investigated in this study. Furthermore, the discrepancy in the findings could be due to different methods and neuropsychological tools used to screen for cognitive impairments and quality of life across studies. Additionally, our sample size was relatively small to capture subtle differences; this is further exacerbated by the fact that the adults with severe cognitive impairments and disability were excluded from this study, a fact that may have compromised our ability to pick up any associations between cognitive impairments and quality of life.

### 4.4 Epilepsy factors and psychological/cognition/psychosocial outcomes

This study shows a limited contribution of epilepsy features to the development of psychopathology in adults with epilepsy. These findings are similar to previous reviews of studies from high-income countries that consistently reported the lack of association of seizure characteristics with psychopathology in epilepsy [44]. However, a childhood study in Kenya found an association between focal seizures and psychopathology [30], but this could be because focal seizures are more common in children than in adults who often report generalized seizures. The limited role of specific epilepsy factors may suggest that the general phenotype of epilepsy, rather than specific features or endophenotypes, contribute to the risk of neurobehavioral impairments. The lack of association could also be explained by psychopathology preceding the onset of epilepsy [45], suggesting shared underlying neurobiological substrates/risk factors, including genetic susceptibility. These results support the possibility that family factors, rather than epilepsy itself per se, may have in part contributed to the psychopathology. Finally, we cannot also overlook the possibility that our sample size may have been too small to pick mild or moderate effects. Larger and more targeted studies may be required to address this question further.

### 4.5 Strengths and limitations

This is among the first studies in SSA that have examined the psychosocial and psychological comorbidities of epilepsy among adults with epilepsy. The tools used in this study were adapted for use in the local population. However, the sample size is small and may have lacked sufficient power to detect differences for some psychosocial measures and to investigate other medical and epilepsy correlates. Additionally, the cases were selected from an epilepsy clinic that attends to patients with more severe epilepsy, and this may have introduced some selection bias. Our focus on participants with low literacy levels may have introduced additional bias.

## 5 Conclusions

This study shows that adults with epilepsy in Kilifi have high levels of depressive symptoms, neurocognitive impairments, and low quality-of-life scores. The situation is even more severe than anticipated since over 30% could not complete neuropsychological tests that were being administered. We further show that psychological comorbidities in PWE affect their quality of life and thus should be addressed. Epilepsy contributes to the burden of mental health and neurocognitive impairments in the community; however, community-based studies are needed to provide precise estimates of these disorders.

The following is the supplementary data related to this article.

Table 1 The impact of cognitive impairment comorbidity on the quality of life of PWE. Table 1
