# Supplementary material for: Cognition, mood and quality-of-life outcomes among low literacy adults living with epilepsy in rural Kenya: A preliminary study
Source: Epilepsy Behav. 2018 Aug;85:45–51. doi: 10.1016/j.yebeh.2018.05.032 (PMC6086937; doi:10.1016/j.yebeh.2018.05.032)
Supplement: Table 1 — The impact of cognitive impairment comorbidity on the quality of life of PWE. [file mmc1.docx]

**Supplementary results**

**Table 1:** **The impact of cognitive impairment comorbidity on the Quality of Life of People with Epilepsy**

|  | **Ravens** (IQ) | | | **Digit span (Memory)** | | |
| --- | --- | --- | --- | --- | --- | --- |
| **Quality of Life Variable** | **Positive for cognitive impairment** | **Negative for cognitive impairment** | **p-value** | **Positive for cognitive impairment** | **Negative for cognitive impairment** | **p-value** |
| Total Quality of Life | 59.7 (17.4) | 57.0 (28.6) | 0.79 | 58.7 (17.2) | 62.7 (24.2) | 0.61 |
| Physical functioning average score | 75.7 (26.1) | 70.0 (30.3) | 0.69 | 76.6 (24.3) | 67.9 (35.3) | 0.43 |
| Role functioning/physical average score | 50.0 (39.5) | 43.8 (42.7) | 0.77 | 47.8 (40.5) | 57.1 (34.5) | 0.57 |
| Role functioning/emotional average score | 43.2 (47.1) | 33.3 (47.1) | 0.69 | 40.2 (46.3) | 52.4 (50.4) | 0.54 |
| Energy/fatigue average score | 50.0 (23.4) | 43.8 (16.5) | 0.61 | 49.9 (22.4) | 47.1 (26.1) | 0.78 |
| Emotional well-being average score | 60.1 (22.4) | 53.0 (30.5) | 0.56 | 57.4 (23.0) | 69.1 (21.6) | 0.22 |
| Social functioning average score | 57.1 (22.5) | 81.3 (12.5) | **0.04** | 59.1 (23.5) | 60.7 (21.0) | 0.88 |
| Pain average score | 54.0 (28.2) | 50.0 (41.0) | 0.80 | 53.1 (29.1) | 56.1 (30.5) | 0.81 |
| General health average score | 54.3 (21.4) | 61.3 (29.5) | 0.56 | 51.5 (21.2) | 72.1 (17.5) | **0.02** |
